# The importance of the rate of pure “attended deaths at home” for objective outcome indicator for assessing the prevalence of home care in Japan

**DOI:** 10.1186/s12199-019-0838-0

**Published:** 2019-12-17

**Authors:** Yasuhiro Kakiuchi, Ryoko Nagao, Eriko Ochiai, Yu Kakimoto, Motoki Osawa

**Affiliations:** Department of Forensic Medicine, Tokai University School of Medicine, Shimokasuya 143, Isehara, Kanagawa 259-1193 Japan

**Keywords:** End-of-life care at home, Super-aging society, Vital Statistics, Regional death investigation system, Police department data

## Abstract

**Background:**

No study has yet been performed on the importance of the rate of pure “attended deaths at home,” excluding examined deaths subjected to a postmortem examination. Therefore, in the present study, we investigated actual state of pure “attended deaths at home,” in order to provide reference data for the future development of end-of-life care at home.

**Methods:**

We performed a detailed survey in Yokohama City according to the type of death, age, and underlying cause of death in cases of home deaths, based on the detailed version of the Vital Statistics Survey Death Forms. Then, we divided deaths occurring in each municipality in Kanagawa Prefecture into two categories: “examined deaths” or “attended deaths,” which were also stratified by the place of death, based on the Vital Statistics, and data on number of death cases subjected to postmortem examination from the Kanagawa Prefectural Police Headquarters.

**Results:**

In 2013, the survey in Yokohama City showed large differences in age distribution and cause of death between examined and attended deaths. In 2014, home deaths accounted for 15.7% of all deaths in the prefecture, whereas the overall proportion of attended deaths at home was 6.9%.

**Conclusions:**

We should utilize the rate of pure “attended deaths at home” for objective outcome indicator.

## Background

Spending the last stage of life in a desired location is an important element of achieving a desirable death for most people [1]. According to a previous study, approximately 50% of Japanese citizens hoped to die at home [2]. Despite this, the proportion of home deaths in Japan was only 13.0% in 2016 [3], which indicates a considerable great discrepancy between what Japanese citizens desire and their actual place of death. In line with the current rate of aging of the Japanese population, the number of deaths in Japan is estimated to reach 1.6 million in 2030. Meanwhile, the number of hospital beds is being increasingly limited to reduce medical care costs in Japan [4]. Currently, there are an insufficient number of hospital beds to accommodate the hundreds of thousands of dying people that will require them in the future. This has caused a concern that “end-of-life care refugees” will emerge as a social problem. To cope with this problem, the Japanese healthcare delivery system must conceive a system whereby people can spend the last stage of their lives where they wish, without fear—not only in hospitals, but also at home.

Several reports on the factors associated with home death have already been published, not only in Japan but also overseas [5–11]. One of them showed that these factors can be broadly classified as disease-associated, personal, and environmental [5].

However, the target of previous studies—that is, the number of people achieving a “home death”—included those who chose to die at home and were receiving home care and other medical services, as well as those who unexpectedly die at home (e.g., solitary deaths) and are subjected to postmortem examination by medical examiners. For example, a survey conducted by the Tokyo Medical Examiner’s Office revealed that 40.7% of home deaths occurring in the wards of Tokyo were solitary deaths [12]. Thus, it would be more appropriate to exclude unexpected deaths, when conducting ecological correlation analysis on the medical factors associated with home death. However, in Japan, data on cases of unexpected deaths subjected to medical examination by medical examiners at home are possessed only by the police and are not disclosed to the public. In addition, these data are not generally shared with other administrative departments (e.g., healthcare and welfare departments). Therefore, in the present study, we investigated the actual state of pure “attended deaths at home,” in order to provide reference data for the future development of healthcare policies.

## Materials and methods

### Study area

The study area was Kanagawa Prefecture, which is adjacent to Tokyo Metropolis and is located in the southern part of Kanto region in Japan. The prefecture has a total population of over 9 million and includes three big cities, Yokohama, Kawasaki, and Sagamihara. Yokohama City has the second largest population in Japan, following Tokyo.

### Data sources

#### Yokohama City

In 2013, we formally requested use of the detailed version of the Vital Statistics Survey Death Forms of Yokohama citizens from the Ministry of Health, Labour and Welfare (MHLW), in accordance with Article 33 of the Statistics Act. The personal information of the included cases was anonymized, de-identified, and delivered to the Medical Care Bureau of Yokohama City, with details regarding type of death, age, and underlying cause of death. This special survey was conducted only in Yokohama City, which funded the survey independently and held sole responsibility. In other words, no other municipality in Kanagawa Prefecture apart from Yokohama has conducted a special survey such as this, mainly owing to the budgetary and manpower restrictions of each municipality.

#### Kanagawa Prefecture

For the reason stated above, we were unable to obtain detailed information about type of death, age, and underlying cause of death from any other municipality in Kanagawa Prefecture except for Yokohama. Therefore, in 2014, we obtained data on the number of deaths that occurred in each municipality except for Yokohama of Kanagawa Prefecture according to the place of death from the Vital Statistics Survey, which are publicly available at the portal site of the Census and Statistics Department of the Japanese government (E-STAT). In addition, we retrieved data on the number of death cases subjected to postmortem examination according to place of death from the police stations in each region of Kanagawa Prefecture except for Yokohama, with the cooperation of the Coroner Office at the Kanagawa Prefectural Police Headquarters.

### Types of death

Deaths in Japan are generally divided into “attended” and “examined” (i.e., un-attended) deaths, based on the type of postmortem examiner. When the cause of death is found to be an obvious disease, it is regarded as “attended,” and the death certificates are prepared by general physicians. On the other hand, when the cause of death is not immediately found to be a disease, it is not regarded as an attended death but an “examined” one, and the death certificates are prepared by medical examiners, and there is also a police investigation. In other words, cases in which medical examiners prepared death certificates are regarded as “examined” deaths, whereas cases in which general physicians prepared the certificates are regarded as “attended” deaths (i.e., pure natural death). We further sub-divided examined deaths into “pure unnatural deaths” (i.e., homicide, suicide, accidental deaths [e.g., falling and drowning], and deaths from unknown causes) and “suspected unnatural deaths” (i.e., deaths subjected to postmortem examination because it is either a solitary one or because of another reason; the cause of these deaths is ultimately determined to have been a disease).

### Statistical analysis

The detailed version of the Vital Statistics Survey Death Forms of Yokohama citizens in 2013 were divided into examined (subdivided into pure and suspected unnatural) and attended deaths as stated above. The type of death, age, and underlying cause of death were cross-tabulated in cases of home deaths. Pearson’s *χ*^2^ test was used to evaluate differences in the proportion of deaths from “cardiac disease” or “malignant neoplasm” between the attended and suspected unnatural death groups among the elderly aged 75 and above. The significance level was defined at 5%. SPSS Statistics 19 (IBM, Tokyo, Japan) was used for all the analyses.

We divided deaths occurring in each municipality in Kanagawa Prefecture in 2014 into two categories: “examined deaths” and “attended deaths,” according to the definition as stated above. Death cases were also stratified by the place of death. The “proportion of home deaths” was defined as the ratio of the number of home deaths to the total number of deaths, while the “proportion of attended deaths at home” was defined as the ratio of the number of attended deaths at home to the total number of deaths. Because some of the police stations within the region had jurisdiction over several municipalities, these relevant municipalities were treated as one group. We calculated the average and standard deviation in the proportion of both home deaths and attended deaths at home.

## Results

Table 1 displays the results of the cross-tabulation of the types of death, age, and underlying causes of death among Yokohama citizens who died at home (*n* = 4,847) from among all citizens who died in 2013 (*n* = 31,573). As shown in Table 1, there were 2305 attended deaths and 2542 examined deaths. When we further sub-divided examined deaths, we observed 652 “pure unnatural deaths” and 1890 “suspected unnatural deaths.” Table 1 also shows the age distribution of the three groups of the deaths (pure unnatural, suspected unnatural, and attended). Although elderly people aged 75 or older accounted for approximately 50% or less of the unnatural and suspected unnatural death groups, they accounted for nearly 80% of the attended death group, with a significant difference (*p* < 0.05). Finally, Table 1 shows the detailed underlying causes of death by age group among two of the three groups (because all deaths in the unnatural death group would naturally include exogenous deaths and deaths from unknown causes, we do not show the detailed underlying causes of deaths for this group). The most common underlying cause was “cardiac disease,” accounting for approximately 54%, with a significant difference (*p* < 0.05) in the suspected unnatural death group, while it was “malignant neoplasm,” accounting for approximately 46%, with a significant difference (*p* < 0.05) in the attended death group.

**Table 1:** Breakdown of home deaths in Yokohama City in 2013 by age group and underlying cause of death in attended, pure and suspected unnatural death

Type of death		Examined death		Attended death (*n* (%))
		Pure unnatural death (*n* (%))	Suspected unnatural death (*n* (%))	
Age group	0–14 years	4 (0.6)	5 (0.3)	1 (0.0)
	15–64 years	264 (40.5)	476 (25.2)	187 (8.1)
	65–74 years	137 (21.0)	479 (25.3)	337 (14.6)
	≧ 75 years	247 (37.9)	930 (49.2)	1780 (77.2)
	Total	652 (100.0)	1890 (100.0)	2305 (100.0)
Cause of death	Infection and parasitosis		2 (0.1)	7 (0.3)
	Malignant neoplasm		97 (5.1)	1054 (45.7)
	Cardiac disease		1019 (53.9)	180 (7.8)
	Cerebrovascular disease		237 (12.5)	49 (2.1)
	Other cardiovascular diseases		49 (2.6)	22 (1.0)
	Pneumonia		29 (1.5)	162 (7.0)
	Other respiratory diseases		96 (5.1)	108 (4.7)
	Digestive system disease		140 (7.4)	41 (1.8)
	Renal disease		3 (0.2)	49 (2.1)
	Senility		57 (3.0)	530 (23.0)
	Other causes		144 (7.6)	91 (3.9)
	Unknown		17 (0.9)	12 (0.5)
	Total		1890 (100.0)	2305 (100.0)

Table 2 shows the number of deaths according to the place and type of death (examined death vs. attended death) in each municipality in Kanagawa Prefecture, in 2014. In 2014, home deaths accounted for 15.7% of all deaths in the prefecture, while hospital or clinic deaths accounted for 73.9% and deaths at other places (e.g., nursing homes) for 10.4%. When the proportion of home deaths was measured by municipality, it was highest, at 22.9%, in Yokosuka City and lowest, at 10.8%, in Naka district (comprising Oiso and Ninomiya town). The standard deviation of this proportion was 2.7% and coefficient of variation (the ratio of the standard deviation to the mean), 0.172. The overall proportion of attended deaths at home in Kanagawa Prefecture was 6.9%. According to municipality, the proportion of attended deaths at home was highest in Yokosuka City, at 15.0%, and lowest in Zama City, at 2.0%. The standard deviation was 3.5% and coefficient of variation, 0.507.

**Table 2:** The number of deaths in 2014 according to place of death in each municipality in Kanagawa Prefecture

City/county	Place of death	Total		Home		Hospital/clinic (Note 6)		Others (e.g., nursing home (Note 7))	
		Total (*n*)	Attended death (*n* (%))	Subtotal (*n* (%))	Attended death (*n* (%))	Subtotal (*n* (%))	Attended death (*n* (%))	Subtotal (*n* (%))	Attended death (*n* (%))
Yokohama City		30,038	26,661 (88.8)	4891 (16.3)	2148 (7.2)	21,817 (72.6)	21792 (72.5)	3330 (11.1)	2721 (9.1)
Kawasaki City		10,134	8945 (88.3)	1698 (16.8)	740 (7.3)	7428 (73.3)	7415 (73.2)	1008 (9.9)	790 (7.8)
Sagamihara City		5459	4801 (87.9)	667 (12.2)	132 (2.4)	4374 (80.1)	4370 (80.1)	418 (7.7)	299 (5.5)
Yokosuka City		4592	4154 (90.5)	1052 (22.9)	687 (15.0)	2902 (63.2)	2899 (63.1)	638 (13.9)	568 (12.4)
Kamakura City		1815	1726 (95.1)	290 (16.0)	213 (11.7)	1289 (71.0)	1288 (71.0)	236 (13.0)	225 (12.4)
Zushi City		612	573 (93.6)	92 (15.0)	59 (9.6)	452 (73.9)	452 (73.9)	68 (11.1)	62 (10.1)
Miura City		623	570 (91.5)	93 (14.9)	47 (7.5)	465 (74.6)	465 (74.6)	65 (10.4)	58 (9.3)
Miura-county		329	307 (93.3)	65 (19.8)	46 (14.0)	207 (62.9)	207 (62.9)	57 (17.3)	54 (16.4)
Fujisawa City		3192	2899 (90.8)	458 (14.3)	215 (6.7)	2359 (73.9)	2356 (73.8)	375 (11.7)	328 (10.3)
Chigasaki City	(Note 1)	2278	2053 (90.1)	315 (13.8)	124 (5.4)	1776 (78.0)	1776 (78.0)	187 (8.2)	153 (6.7)
Hiratsuka City		2245	2003 (89.2)	360 (16.0)	166 (7.4)	1712 (76.3)	1708 (76.1)	173 (7.7)	129 (5.7)
Hadano City		1357	1217 (89.7)	198 (14.6)	90 (6.6)	1025 (75.5)	1019 (75.1)	134 (9.9)	108 (8.0)
Isehara City		799	712 (89.1)	104 (13.0)	39 (4.9)	625 (78.2)	625 (78.2)	70 (8.8)	48 (6.0)
Naka County		638	583 (91.4)	69 (10.8)	28 (4.4)	500 (78.4)	499 (78.2)	69 (10.8)	56 (8.8)
Atsugi City	(Note 2)	2097	1843 (87.9)	284 (13.5)	79 (3.8)	1563 (74.5)	1560 (74.4)	250 (11.9)	204 (9.7)
Yamato City	(Note 3)	2443	2202 (90.1)	290 (11.9)	93 (3.8)	1990 (81.5)	1990 (81.5)	163 (6.7)	119 (4.9)
Ebina City		863	772 (89.5)	100 (11.6)	32 (3.7)	699 (81.0)	698 (80.9)	64 (7.4)	42 (4.9)
Zama City		1080	926 (85.7)	148 (13.7)	22 (2.0)	858 (79.4)	858 (79.4)	74 (6.9)	46 (4.3)
Odawara City	(Note 4)	2736	2422 (88.5)	378 (13.8)	134 (4.9)	2090 (76.4)	2088 (76.3)	268 (9.8)	200 (7.3)
Minamiashigara City	(Note 5)	1,057	939 (88.8)	148 (14.0)	58 (5.5)	822 (77.8)	822 (77.8)	87 (8.2)	59 (5.6)
All of Kanagawa		74,387	66,308 (89.1)	11,700 (15.7)	5152 (6.9)	54,953 (73.9)	54,887 (73.8)	7734 (10.4)	6269 (8.4)

Note 1: Including Koza county (Samukawa town)

Note 2: Including Aiko county (Aikawa town and Kiyokawa village)

Note 3: Including Ayase City

Note 4: Including Ashigarakami county (Nakai, Oi, Matsuda, Yamakita, and Kaisei town)

Note 5: Including Ashigarashimo county (Hakone, Manazuru, and Yugawara town)

Note 6: “Hospital” means a facility for the hospitalization of not less than 20 patients, where physicians or dentists carry out medical practices or dental practices for the public or other specific groups of people, according to Medical Care Act in Japan. “Clinic” means a facility with no in-patient capacity, or a facility for the hospitalization of no more than 19 patients, where physicians or dentists carry out medical practices or dental practices for the public or other specific groups of people

Note 7: “Nursing home” means geriatric health services facility providing care for bathing, bodily waste elimination, meals, etc., and other care for performing daily activities, according to Long-Term Care Insurance Act in Japan

## Discussion

In the present study, we performed an analysis of the attributes of examined and attended deaths in Yokohama City (the municipality with the largest population in Kanagawa Prefecture) in 2013 and observed large differences in age distribution and cause of death between the two types of death. Then, death cases collected according to the place of death in each municipality in Kanagawa Prefecture in 2014 were divided according to whether or not they had been subjected to a postmortem examination, in order to accurately ascertain the number of people who were cared for at home and achieved a home death.

We observed large differences in age distribution and cause of death between examined and attended deaths. Specifically, elderly people aged 75 or older accounted for approximately 80% of attended deaths, and the underlying cause of approximately 50% of these deaths was malignant neoplasm. Approximately 75% of the examined deaths were ultimately determined to be deaths from disease, and the most common causes (about 70%) were cardiac or cerebrovascular diseases. Because the attended and examined deaths greatly differed in their attributes, as described above, it is questionable whether both of them should be considered as types of home death or used as indicators of home care in an effort to develop evidence-based healthcare policies in each municipality. Meanwhile, because the Vital Statistics Survey Death Forms (which were used as data for Table 1) lack information on family composition and others, it is unclear what percentage of examined deaths were solitary deaths. However, a previous study revealed that solitary deaths accounted for about 40.7% of all home deaths in the wards of Tokyo [12].

The overall proportion of home deaths in Kanagawa Prefecture in 2014 was 15.7%, which was higher than the national average of 12.8%. The proportion of attended deaths at home—that is, when we excluded examined deaths—was less than half of the overall proportion, at 6.9%. The highest proportion of home deaths was observed in Yokosuka City (22.9%), which was approximately twice as high as the lowest proportion (observed in Naka district, 10.8%). The disparity between the highest and lowest proportion of attended deaths at home (Yokosuka City, at 15.0%, and Zama City, at 2.0%, respectively) widened to approximately 7.5 times, which was reflected in the increase in coefficient of variation. Presently, the MHLW employs the proportion of home death as but one objective outcome indicator for assessing the prevalence of home care in each municipality [13]. However, at least in Kanagawa Prefecture, more than half of home death cases were examined ones, as stated above. This calls into question the accuracy of using the proportion of home death as an indicator of home care in the region. The fact that the MHLW is not able to further specify home deaths is attributed to the process of aggregating data from the Vital Statistics Survey Death Forms, which contains no detailed information regarding postmortem examination. Such information would be extremely useful not only for analyzing the present status of home care, but also for ascertaining the real number of solitary deaths, which are expected to rapidly increase in the near future. Thus, the process of data aggregation needs to be improved immediately. If this cannot be done immediately, an alternative measure might be instituted by building a system in which police departments (who currently collect data on examined deaths) and the healthcare and welfare department (who are in charge of mortality statistics) share their data through close cooperation in each municipality.

As stated above, large differences in age distribution and cause of death between examined and attended deaths were observed. In addition, the proportion of attended deaths at home was less than half of the overall proportion. Therefore, both examined and attended deaths should be considered separately as different types of home death. Additionally, the rate of pure “attended deaths at home” should be used as an objective outcome indicator in assessing the prevalence of home care. A recent innovative research analyzed the medical care provided to end-of-life elderly patients, based on the National Database of Health Insurance Claims and Specific Health Checkups of Japan (NDB) [14]. The spread of utilization of NDB will support the imperfection of the vital statistics stated above and elucidate the actual status of home care in Japan.

Despite these novel findings, the present study has several limitations. First, because it targeted only cases in Kanagawa Prefecture, further studies are needed to determine whether the obtained conclusion is applicable to other regions of Japan. Next, regarding the data on examined deaths used in the present study, as described in the “Materials and methods” section of this paper and the notes of Table 2, we were unable to obtain individual municipal data from some small municipalities because their data were aggregated. However, because these small municipalities in Kanagawa Prefecture include a region with the highest proportion of home deaths in the prefecture—Yamakita town (24.3%) [15]—the present conditions of attended deaths at home in the individual municipalities making up this region also need to be further examined in detail. For this reason, prefectural police departments in not only Kanagawa Prefecture but also other prefectures in Japan need to realize the public health significance of the data that they store and manage on examined deaths and to review the methods of data management in terms of cooperation with the healthcare and welfare departments of each municipality.

## Conclusion

In the present study, we observed large differences in age distribution and cause of death between the examined and attended deaths. Then, death cases collected according to the place of death were divided according to whether or not they had been subjected to a postmortem examination. Consequently, the proportion of attended deaths at home was less than half of the overall proportion. Therefore, we should utilize the rate of pure “attended deaths at home” for objective outcome indicator for assessing the prevalence of home care.

## Data Availability

The detailed version of the Vital Statistics Survey Death Forms of Yokohama citizens in 2013 was provided from the Ministry of Health, Labour and Welfare (MHLW), in accordance with Article 33 of the Statistics Act. The personal information of the included cases was anonymized, de-identified, and delivered to the Medical Care Bureau of Yokohama City. The data on the number of deaths that occurred in each municipality of Kanagawa Prefecture in 2014, according to the place of death is publicly available at the portal site of the Census and Statistics Department of the Japanese government (E-STAT). The data on the number of death cases subjected to postmortem examination according to place of death was retrieved from the police stations in each region of Kanagawa Prefecture, with the cooperation of the Coroner Office at the Kanagawa Prefectural Police Headquarters. The datasets are not publicly available due to the criminal policy in Japan.

## Abbreviations

E-STAT: Portal site of the Census and Statistics Department of the Japanese government
MHLW: Ministry of Health, Labour and Welfare
OECD: Organization for Economic Co-operation and Development
